# Incivility in medical education: a scoping review

**DOI:** 10.1186/s12909-022-03988-2

**Published:** 2023-01-12

**Authors:** Laura E. Abate, Larrie Greenberg

**Affiliations:** School of Medicine & Health Sciences, The George Washington University, 2300 Eye St NW, Washington, DC 20037 USA

**Keywords:** Incivility, Bullying, Harassment, Abuse, Discrimination, Medical education, Medical school, Residency, Residents, Faculty

## Abstract

Incivility in the workplace, school and political system in the United States has permeated mass and social media in recent years and has also been recognized as a detrimental factor in medical education. In this scoping review, we use the term incivility to encompass a spectrum of behaviors that occur across the continuum of medical education, and which include verbal abuse including rude or dismissive conduct, sexual and racial harassment and discrimination, and sexual and physical assault. We identified research on incivility involving medical students, residents and fellows, and faculty in North America to describe multiple aspects of incivility in medical education settings published since 2000. Our results reinforce that incivility is likely under-reported across the continuum of medical education and also confirmed incidences of incivility involving nursing personnel and patients, not emphasized in previous reviews. The authors suggest a zero-tolerance national policy if this problem is to be resolved.

## Background

Incivility in workplaces and in schools in the United States has permeated mass media and social media in recent years [1]. The recent United States presidential elections were notable for their tone of anger, negativity, and incivility. In the fall of 2017, a series of sexual harassment accusations and admissions in the entertainment industry demonstrated the frequency and serial nature of this type of harassment [2]. Hazing and sexual abuse in athletics have also been reported in high school, college, and Olympic-level sports [3, 4]. In other words, incivility is pervasive in the United States.

Incivility has been addressed by reviews on incivility in the health sciences and in the medical education literature [5–7]. A general definition of incivility implies disregard and insolence for others, causing an atmosphere of disrespect, conflict, and stress [8]. Leape et al. published back-to-back commentaries that addressed the nature and causes of disrespectful physician behavior followed by one calling for creating a culture of respect [9, 10]. These authors identified a dysfunctional, hierarchical culture in academic medicine that seems to be self-perpetuating and emphasized how this impacts not only on individuals and the institution but importantly affects patient safety. For this review, we will build on the previous work on the subject and also frame the issues more broadly by using the term incivility to describe a spectrum of behaviors including rude or dismissive conduct; legally recognized forms of incivility including sexual harassment or racial discrimination; and criminal acts including physical and sexual assault. While we are defining incivility broadly, it does not include behaviors such as being late or not knowing appropriate information about one’s patients, which more closely align with non-professional behavior.

Incivility across the continuum of medical education has likely been a problem since the origins of organized medicine in Europe, as reflected in the hierarchical culture that developed when physicians trained in classroom knowledge were more highly regarded than physicians educated via on-the-job training [11]. The European medical system was adapted to the first North American medical schools in the 19^th^ century**,** incorporating many of the characteristics of the hierarchical culture that has led to the hidden curriculum, i.e., what faculty do is not always consistent with what they teach and model [12]. This hierarchy is couched in power and social learning theory, with ethical, personal and lack of empowerment ramifications affecting those on the lower end of the hierarchy, often leading to incivility [13]. Bandura’s social learning theory describes this behavior and suggests that those in power who model unacceptable behavior influence those who are the recipients of this behavior, implying that the system becomes self-perpetuating [13].

Although long accepted as a part of the culture of medicine, incivility has been described and documented as a problem in the health care professions [6, 14–18]. While previous articles have focused on the personnel involved (perpetrators and victims) and the prevalence of incivility worldwide, the major focus of this review is to address the problem in North America. Our scoping review will summarize the current literature identifying those individuals most frequently identified as perpetrators or victims; the types of incivility described; the general settings of the incidents; the impact on the individual and institution, and institutional responses to the issue. The authors will discuss lessons learned from this review and explore root causes underlying civility in the setting of medical education.

## Methods

In preparing this scoping review, we sought to identify articles on incivility in medical education and with this goal in mind, we developed an extensive set of keywords and complex search strategies for PubMed and Scopus (see Appendix) which used multiple representations of incivility including mistreatment, bullying, harassment, and discrimination. To develop the search strategy in PubMed, we identified exemplar articles via title word searches then used those articles to select relevant Medical Subject Headings (MeSH), title, and keyword terms for each concept. The keyword and MeSH searches were combined in the final search strategy. The strategy identifies articles on all types of incivility, including bullying and sexual, racial, and gender-based harassment, across the spectrum of medical training including medical school, residency and fellowship, and among medical school faculty. The search parameters identified articles published in English from 2000 through October 2019.

**Fig. 1:**
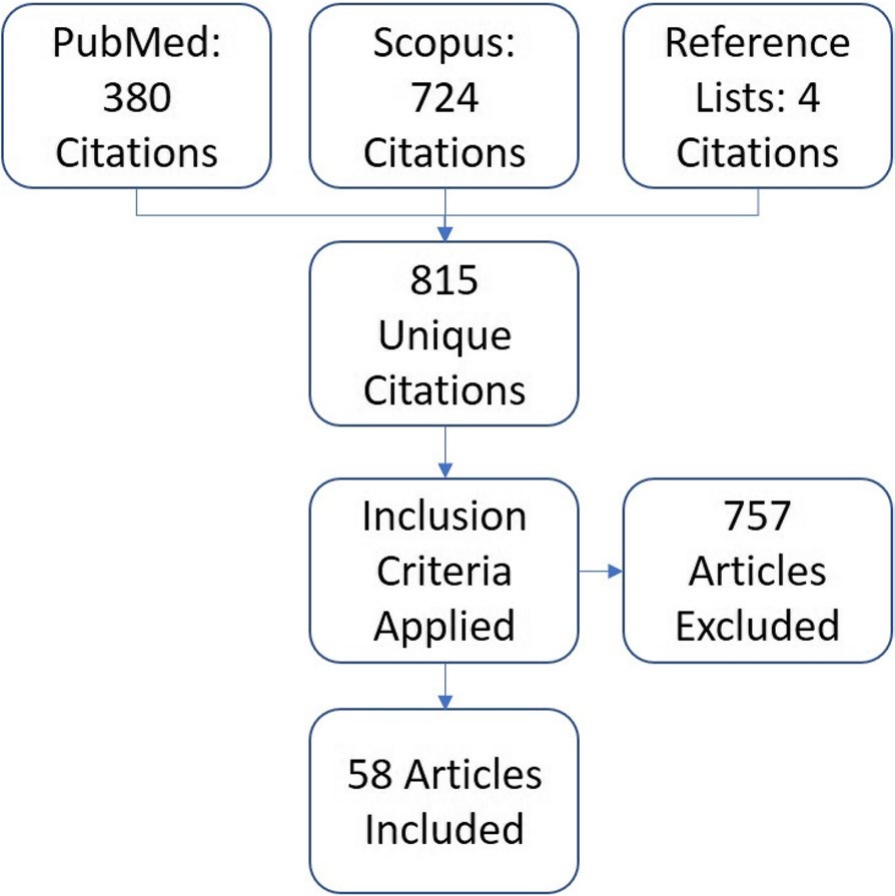
Flow chart describing search and selection process to identify relevant studies

In October 2019, the PubMed search retrieved 380 citations and the Scopus search retrieved 724 citations. After combining the lists and removing duplicate articles, 811 unique articles were identified. Four additional articles were identified via references in this set of articles. For each of the 815 unique articles, each author independently assessed and compared results to determine whether the article met the inclusion criteria. For the 43 articles (8%) for which the authors did not agree regarding the assessment, we discussed each article based on our inclusion criteria to make a final determination. The search and evaluation process identified 58 articles that met the criteria for this study (see Fig. 1 and Table 1).

**Table 1:** Studies included in this review

**Study**	**Study Population**	**Quantitative—Qualitative**	**Types of incivility**	**Outcomes reported**
Power day: Addressing the use and abuse of power in medical training (Angoff et al. 2016, 203–213) [19]	Medical students	Qualitative	Power-based	• Positive and negative examples of uses of power
Professionalism in the teacher-learner relationship in medical schools: mistreatment(Antonelli 2009, 88–89) [20]	Medical students	Quantitative	Verbal, sexual and gender-based	• Perpetrators by professional role • Reasons for not reporting student mistreatment
Awareness of Bullying in Residency: Results of a National Survey of Internal Medicine Program Directors (Ayyala et al. 2018, 209–213) [21]	Residents	Quantitative	Less than 1/3 respondents reported bullying, with verbal > physical abuse	• Decreased performance and depression
Perceived Bullying Among Internal Medicine Residents (Ayyala, Rios, and Wright 2019, 576–578) [22]	Residents	Quantitative	Bullying and verbal abuse	•Concerns about burnout, decreased performance, depression, weight change/nutrition
"I'm too used to it": a longitudinal qualitative study of third year female medical students' experiences of gendered encounters in medical education(Babaria et al. 2012, 1013–1020) [23]	Medical students	Qualitative	Sexual and gender-based	•Perpetrators via student-patient and student-supervisor relationships •Impact on student self-image •Students’ adaptations to managing inappropriate behavior
The learning environment in the obstetrics and gynecology clerkship: an exploratory study of students' perceptions before and after the clerkship (Baecher-Lind, Chang, and Blanco 2015, 27,273) [24]	Medical students	Qualitative	Verbal, sexual, physical	•Perceptions of mistreatment pre-clerkship and post-clerkship
Underlying mechanisms of mistreatment in the surgical learning environment: A thematic analysis of medical student perceptions (Brandford et al. 2018, 227–232) [25]	Medical students	Qualitative	Exclusion form medical team, obstruction of student learning, not being fair or respectful, exploiting student vulnerability, assigning non-educational tasks	•Students need to be encouraged to report mistreatment
Clerkship-Specific Medical Student Mistreatment (Breed et al. 2018, 477–482) [26]	Medical students	Quantitative	Public humiliation, gender discrimination	• Occurs more often in the operating room • Surgery > Ob > Internal Medicine
Sexual Harassment in Ophthalmology: A Survey Study (Cabrera et al. 2019, 172–174) [27]	Faculty and residents	Quantitative	Sexual harassment	• Interfered with the ability to work • 15% changed jobs and/or careers • American Academy of Ophthalmology has established zero tolerance policy for sexual harassment
Sexual Harassment in Radiology (Camargo, Liu, and Yousem 2017, 1094–1099) [28]	Faculty and residents	Quantitative	Sexual harassment	• Needs to be transparency of reporting • Females less likely to report • Females > males
A "ton of feathers": Gender discrimination in academic medical careers and how to manage it (Carr et al. 2003, 1009–1018) [29]	Faculty	Qualitative Quantitative	Gender-based	• Effect of gender discrimination on academic medicine career
Mistreatment and the learning environment for medical students on general surgery clerkship rotations: What do key stakeholders think? (Castillo-Angeles et al. 2017, 307–312) [30]	Medical students	Qualitative	Mistreatment, neglect, unclear expectations, not integrating into the team, negative attitudes about lack of knowledge, humiliation, sexual harassment	• Focus on learning environment
Bullying in the American graduate medical education system: A national cross-sectional survey (Chadaga, Villines, and Krikorian 2016, e0150246) [31]	Residents	Quantitative	Verbal	• Most common types of bullying and association of personal attributes with risk of bullying • Perpetrators by professional role • Impact on health
Workplace Bullying of Urology Residents: Implications for the Patient and Provider (Chowdhury, Husainat, and Suson 2019, 30–35) [32]	Residents	Quantitative	98% reported bullying	• Perceptions is that this had negative effects on personal behavior and patient care
Intraoperative Disruptive Behavior: The Medical Student's Perspective (Chrouser and Partin 2019, 1231–1240) [33]	Medical students	Qualitative	Verbal abuse in the operating room	• Personal and team consequences with a result of decreased work
Exploring medical students' barriers to reporting mistreatment during clerkships: a qualitative study (Chung et al. 2018, 1478170) [34]	Medical students	Qualitative	Mistreatment, verbal and physical abuse	• Barriers to reporting: fear of reprisal, perceptions that medical culture includes mistreatment, the difficulty of reporting subtler forms of mistreatment, can damage teacher-students relationship, reporting process cumbersome and is reporting beneficial
The prevalence of medical student mistreatment and its association with burnout (Cook et al. 2014, 749–754) [14]	Medical students	Quantitative	Mistreatment	• Frequency of mistreatment by faculty and residents • Prevalence of burnout in medical students by degree of reported mistreatment
A Survey Study of Resident Experiences of Sexual Harassment during Dermatology Training (DeWane et al. 2019) [35]	Residents	Quantitative	Sexual harassment by patients, faculty and fellow residents	• Greater in females than males • Sexist hostility and gender harassment
Feedback matters: the impact of an intervention by the dean on unprofessional faculty at one medical school (Dorsey, Roberts, and Wold 2014, 1032–1037) [36]	Medical students	Quantitative	Verbal	• Unprofessional faculty behaviors most frequently mentioned by graduating medical students
An Empirical National Assessment of the Learning Environment and Factors Associated with Program Culture (Ellis et al. 2019, 585–592) [37]	Residents	Quantitative	Verbal and physical abuse, gender discrimination, sexual harassment, burnout	• Wellness inversely proportional to duty hours • Program culture determined by wellness and negative exposures
Medical student abuse from multiple perspectives (Elnicki, Ogden, and Wu 2007, 153–158) [38]	Medical students Residents Faculty Nurses	Quantitative	Verbal, ethnic, sexual	• Agreement regarding if scenarios represented abuse and if type of behavior should be reported
Screening for Harassment, Abuse, and Discrimination among Surgery Residents: An EAST Multicenter Trial (Fitzgerald et al. 2019, 456–461) [39]	Residents	Quantitative	Sexual harassment	• Impact on learning climate, promoting anger, frustration and embarrassment
Eradicating medical student mistreatment: a longitudinal study of one institution's efforts (Fried et al. 2012, 1191-1198) [40]	Medical students	Quantitative	Verbal, power-based, sexual, ethnic, physical	• Types of mistreatment before and after adoption of ‘Statement Supporting an Abuse-Free Academic Community’ • Perpetrators by professional role including patients
Association Between Perceived Medical School Diversity Climate and Change in Depressive Symptoms Among Medical Students: A Report from the Medical Student CHANGE Study (Hardeman et al. 2016, 225–235) [41]	Medical students	Quantitative	Negative role modeling, mistreatment, negative racial climate, ignored, humiliated	• Increased depressive symptoms related to negative behavior • Call for creating an institutional climate that is inclusive, fair and equitable
Formation of medical student professional identity: categorizing lapses of professionalism, and the learning environment (Hendelman and Byszewski 2014, 139) [42]	Medical students	Quantitative	Verbal, power-based, cultural or religious, sexual	• Types of professionalism lapses witnessed by pre-clerkship and clerkship medical students • Prevalence of witnessing professionalism lapse during medical school • Perpetrators of professional lapses during pre-clerkship and clerkship
'Am I being over-sensitive?' Women's experience of sexual harassment during medical training (Hinze 2004, 101–127) [43]	Residents	Quantitative Qualitative	Sexual	• Experiences of and discomfort with specific sexist treatment variables • Settings by specialty
Patterns of Disrespectful Physician Behavior at an Academic Medical Center: Implications for Training, Prevention, and Remediation (Hopkins et al. 2018, 1679–1685) [44]	Medical staff	Quantitative	Disrespectful behavior	• For faculty, this was highest in the operating room; for trainees this was highest on the med/surg units • Higher in procedural areas than non-procedural • Males > females, medicine > surgery
Tracking Student Mistreatment Data to Improve the Emergency Medicine Clerkship Learning Environment (House et al. 2018, 18–22) [45]	Medical students	Qualitative	Ignored or marginalized, treated unprofessionally	• Need for data over time and faculty development to confront issue
Professionalism in plastic surgery: attitudes, knowledge, and behaviors in medical students compared to surgeons in training and practice–one, but not the same (Hultman and Wagner 2015, S247-54) [46]	Medical students Faculty	Quantitative	Verbal, physical, sexual	• Types of unprofessional behavior witnessed by medical students and faculty • Prevalence of observation of unprofessional behavior by health care personnel
Impact of a program to diminish gender insensitivity and sexual harassment at a medical school (Jacobs, Bergen, and Korn 2000, 464–469) [47]	Faculty	Quantitative	Sexual	• Prevalence of sexually harassing behaviors during multi-year program to educate faculty on gender issues and diminish sexual harassment
Sexual harassment and discrimination experiences of academic medical faculty (Jagsi et al. 2016, 2120–2121) [48]	Faculty	Quantitative	Sexual, gender-based	• Types of sexual harassment experienced • Prevalence of gender-based bias or advantage and sexual harassment • Effect of gender-based bias on professional advancement
Identifying Medical Student Mistreatment in the Obstetrics and Gynecology Clerkship (Kappy et al. 2019) [49]	Medical students	Quantitative Qualitative	Treated as ‘stupid’; Discouraged from asking questions; ignored; marginalized; nonprofessional behavior	• Students reported a high rate of mistreatment • Conclusion is to improve the learning environment
Reported Mistreatment During the Surgery Clerkship Varies by Student Career Choice (Kemp et al. 2018, 918–923) [50]	Faculty and residents	Quantitative	Verbal/physical abuse, negative physician attitudes, sexual harassment, gender discrimination, public humiliation	• Mistreatment appears to be improving • Career choice of students correlated with perceptions of mistreatment
Verbal aggressiveness among physicians and trainees (Lazarus et al. 2016, 756–760) [51]	Medical students Residents Faculty	Quantitative	Verbal, physical	• Prevalence bullying in medical school • Sources of trainee bullying • Settings • Association of Infante Verbal Aggressiveness Scale (IVAS) with specialties, attending and trainee characteristics
Post-traumatic Stress Disorder in Resident Physicians (Lo et al. 2019, e4816) [52]	Residents	Quantitative	Bullying, violence	• Concerns about post-traumatic stress disorder
Medical student mistreatment: understanding 'public humiliation' (Markman et al. 2019) [53]	Medical students	Qualitative	Public humiliation	• May be amenable to intervention through teaching faculty the importance of orientation and clear communication of intent
Learning about medical student mistreatment from responses to the medical school graduation questionnaire (Mavis et al. 2014, 705–711) [16]	Medical students	Quantitative	Verbal, physical, sexual, racial/ethnic, sexual orientation	• Prevalence of types of mistreatment • Perpetrators of mistreatment • Awareness regarding school policies; reporting practices
Perception of Shame in otolaryngology-head and neck surgery training (McMains et al. 2015, 786–790) [54]	Residents Faculty	Quantitative	Shaming	• Prevalence of shaming • Sources of shaming • Settings • Effects of shaming on individual
The Culture of Academic Medicine: Faculty Behaviors Impacting the Learning Environment (Moutier et al. 2016, 912–918) [55]	Faculty	Quantitative	Derogatory behavior, anger, hostile email and verbal communication, bullying, sexual harassment	• Decreased work production, jeopardized patient care • Strategies should be directed to improve the learning climate
Interns' experiences of disruptive behavior in an academic medical center (Mullan, Shapiro, and McMahon 2013, 25–30) [56]	Residents	Quantitative	Verbal, gender-based, racial, physical	• Types of disruptive behavior • Perpetrators of disruptive behavior
A qualitative study of gender differences in the experiences of general surgery trainees (Myers et al. 2018, 127–134) [57]	Residents	Qualitative	Lewd remarks, interpreting other’s behavior as aggressive Females affected more than males	• Women perceive lack of mentorship, discomfort, pressure to accept/participate in unprofessional behavior, difficulty completing tasks, more barriers during training that interfere with self-identification as surgeons versus men
Medical students' perception of lesbian, gay, bisexual, and transgender (LGBT) discrimination in their learning environment and their self-reported comfort level for caring for LGBT patients: a survey study (Nama et al. 2017, 1368850) [58]	Medical students	Quantitative	Negative comments about sexual orientation, jokes rumors, bullying, harassment	• This is peer-on-peer incivility and based on perceptions • Anti-LGBT discrimination and heterosexism noted by peers, although not affecting the care of LGBT patients • This discrimination presents a difficult learning environment for these students
Gender discrimination and sexual harassment in medical education: perspectives gained by a 14-school study (Nora et al. 2002, 1226–1234) [59]	Medical students	Quantitative	Sexual, gender-based	• Prevalence of gender discrimination/sexual harassment • Settings by specialty
Faculty self-reported experience with racial and ethnic discrimination in academic medicine (Peterson et al. 2004, 259–265) [60]	Faculty	Quantitative	Racial/ethnic	• Prevalence by race • Effect on career satisfaction, job stability, professional confidence
Sexual Harassment of Canadian Medical Students: A National Survey (Phillips et al. 2019, 15–20) [61]	Medical students	Quantitative	Sexual harassment	• Predominantly by patients, then fellow students and faculty • Perpetrators all male and 98% victims female • Resulted in shame and self-blame of victims • Silence to this problem not acceptable
To the point: undergraduate medical education learner mistreatment issues on the learning environment in the United States (Pradhan et al. 2019) [62]	Medical students	Quantitative	Public humiliation, sexual remarks	• Resulted in negative learning climate • Remedies need to be directed to faculty, residents and students with a zero tolerance policy
Supervisor-trainee relationship boundaries in medical education (Recupero et al. 2005, 484–488) [63]	Residents	Quantitative	Supervisor-trainee boundaries, sexual	• Types of boundary violations • Perpetrators by supervisory role
Patterns and predictions of resident misbehavior–a 10-year retrospective look (Resnick et al. 2006, 418–425) [64]	Residents	Quantitative	Verbal, physical	• Types of mistreatment • Victims • Perpetrators by surgical specialty,
Impact and implications of disruptive behavior in the perioperative arena (Rosenstein and O'Daniel 2006, 96–105) [65]	Residents Faculty Nurses and other perioperative staff	Quantitative	Verbal, physical	• Types of disruptive behavior • Perpetrators by professional role • Effect on individual and clinical care
Workplace bullying of general surgery residents by nurses (Schlitzkus et al. 2014, e149-54) [66]	Residents	Quantitative	Verbal	• Types of bullying
Workplace violence and harassment against emergency medicine residents (Schnapp et al. 2016, 567–573) [67]	Residents	Quantitative	Physical abuse and verbal harassment by patients, sexual harassment	• 75% felt safe at work • Need to understand the prevalence of workplace violence for prevention
Training-related harassment and drinking outcomes in medical residents versus graduate students (Shinsako, Richman, and Rospenda 2001, 2043–2063) [68]	Residents	Quantitative	Verbal, sexual	• Types of harassment • Victims’ gender and Michigan Alcoholism Screening Test (MAST) scores
Mistreatment of medical students in the third year may not be the problem (Slavin and Chibnall 2017, 891–893) [69]	Medical students	Quantitative	Working with unhappy residents and attending physicians, ignored, feeling incompetent, unfair evaluations	• Assess underlying reasons why attending physicians and residents are unhappy, leading to burnout
Does students' exposure to gender discrimination and sexual harassment in medical school affect specialty choice and residency program selection? (Stratton et al. 2005, 400–408) [70]	Medical students	Quantitative	Gender-based, sexual	• Setting by specialty • Influence on specialty choice and residency rankings
Emergency medicine resident wellness: Lessons learned from a national survey (Taher et al. 2018, 721–724) [71]	Residents	Quantitative	Verbal harassment	• Results were falling asleep at the wheel, motor vehicle accidents, stress, fatigue, mood swings, suicidal ideation • Call for more investigation with validated tools for stakeholders
Prevalence of Horizontal Violence Among Emergency Attending Physicians, Residents, and Physician Assistants (Volz et al. 2017, 213–218) [72]	Faculty, residents, physician assistants	Quantitative	Verbal aggression, demeaning remarks, not respected re: professional decisions, subject of rumors, isolation	• 9% stated this impacted their health and the care of their patients
Gender-based discrimination is prevalent in the integrated vascular trainee experience and serves as a predictor of burnout (Wang et al. 2019) [73]	Residents	Quantitative	Public humiliation, others taking credit for one’s work, assigned tasks as punishment, physical violence, gender/race/ethnicity mistreatment, sexual harassment	• Negative workplace experience • Affected relationships with staff, women > men
Resident bullying in diagnostic radiology (Wolfman and Parikh 2019, 47–52) [74]	Residents	Quantitative	Bullying	• 28% experienced bullying and 33% witnessed bullying

Our inclusion criteria specified that each article must address at least one type of incivility based on a broad definition, which includes verbal harassment, sexual harassment, gender discrimination, racial harassment, and physical and sexual assault, and that the incivility occur during undergraduate or graduate medical education or among medical school faculty. The inclusion criteria also specified that each article must discuss and provide data on one or more aspect of incivility such as the type, victims, perpetrators, the setting, and/or the impact of incivility on the individual or the institution. We included articles that describe incivility in medical education in North America and excluded other geographic areas due to possible differences in the structure and culture across the continuum of medical education. We also excluded studies that addressed incivility in health disciplines such as nursing and other health sciences and focused solely on medical education.

Our final set of studies that met our inclusion criteria included two articles that reported data from the AAMC Medical School Graduation Questionnaire [16, 20]. While both studies reported similar data on perpetrators of incivility, we chose to include both studies in the final set as each provided unique data in other areas and documented different year spans.

## Results

### Types of Incivility

Across studies of medical students, residents, and faculty, verbal abuse is the most frequently described type of incivility [16, 33, 34, 36, 38, 42, 46, 49, 40]. For those studies involving medical students, verbal abuse is described as rude or demeaning behavior [36], arrogant or condescending behavior [36, 42], aggressive questioning [38], bullying or intimidation [46], disruptive behavior [46], poor anger management [46], and public belittling or humiliation [16, 26, 53, 62]. Sexual harassment and sexual misconduct toward medical students was also documented in multiple reports [16, 38, 42, 46, 53, 61, 40] and includes experiences which range from inappropriate flirting to sexual assault [23, 24], sexual mistreatment (sexual favors for grades, unwanted sexual advances, sexist remarks or names [30, 61, 62] and gender-based mistreatment (denial of opportunities/rewards, lower evaluations or grades) [16, 26, 58]. Physical abuse has been reported less frequently but does occur [34]. Incivility based on power differences between medical students and their supervisors was also described in multiple studies [19, 42, 40]. Lastly, an area not commonly addressed is when medical students are ignored; not included or integrated into team functions; marginalized; when their learning is obstructed; when they are the subject of rumors; when someone takes credit for their work; when they work with role models who are unhappy; and, when they are neglected [25, 30, 41, 45, 49, 69].

Among studies involving residents, high rates for verbal abuse were reported [21, 37, 50, 56, 64, 71, 72], with specific types of verbal abuse including belittling or undermining work, unjustified criticism or monitoring, and destructive innuendo or sarcasm [31]. Incivility studies on residents differentiated work-related bullying where the most frequent form was being shouted at or made the target of spontaneous anger, from person-related bullying and physical intimidation for which the most common form was being ignored or receiving hostile reaction [21, 22, 32, 52, 66, 74]. Residents also reported undermining behavior by faculty which included belittling, unjustified criticism, exclusion from decision-making, and ignoring patient orders requested by the resident [31, 56, 66, 68]. Residents’ experiences with boundary violations by supervisors were documented as invasions of personal space, inappropriate touching or physical violence, sexual offers, sexual harassment, and offers of better evaluations for sexual favors [37, 39, 50, 52, 57, 63], while their experiences with shaming behavior included ‘banishment’ from the operating room and being yelled at, called names, or threatened [54]. There were also reports of gender and ethnicity discrimination as a form of incivility [35, 37, 50, 73].

For faculty, gender discrimination and sexual harassment were the types of incivility most frequently reported. Gender discrimination was identified as an important factor impeding careers in academic medicine [29] and sexist behavior or remarks was the form of sexual harassment most frequently experienced by faculty [27, 28, 48, 50, 55]. Additional reports described bullying or intimidation as the type of unprofessional behavior most frequently observed by physicians [44, 46, 55], and the elevated rates at which minority and non-minority faculty experienced racial or ethnic remarks or inadequate recognition of their work [60]. Several forms of verbal abuse were also documented including bullying or intimidation, disruptive behavior, and anger management [46, 55, 72]. Studies that reported on mixed populations for students, residents and faculty also documented multiple types of verbal abuse [51, 75].

### Perpetrators

Identification of the perpetrators and victims of incivility in medical education is linked inextricably to the population studied. Residents were the most frequent perpetrators of incivility toward medical students followed closely by faculty [14, 20, 40]. Female medical students identified male faculty and patients as perpetrators of gender-related encounters that included inappropriate flirting and sexual innuendo, inappropriate touching, and solicitation [23]. Pre-clerkship medical students identified their student colleagues as the most common perpetrators of arrogance and cultural or religious insensitivity on other students, while faculty were the most common perpetrators of the abuse of power asymmetries on students [42]. During their clerkship years, medical students identified faculty as the most frequent perpetrators of all types of incivility including arrogance, cultural or religious insensitivity, and abuses of power asymmetries [42].

In studies of residents, faculty were identified as the most common perpetrators of incivility [31, 54, 63]. Interns or first year residents identified nurses as the most recurrent source of disruptive behavior [56]. Nurses and patients closely followed faculty as frequent perpetrators of bullying [31] and patients were also reported as perpetrators of sexual harassment, verbal and physical abuse of faculty and trainees [35, 61, 67].

Although no studies addressed perpetrators of incivility directed towards faculty specifically as a group, several studies reported results for populations comprised of students, residents and attending physicians. Physicians/faculty were identified as the most common perpetrators of disruptive behaviors [75] and bullying [51].

### Victims

In the section on the type of incivility, we document not only the type but those who are victims. Studies that addressed victims provided data on the prevalence of incivility experienced by medical students and addressed the different rates at which male and female medical students experienced incivility. Rates of incivility ranged from 17% of medical students reporting mistreatment [16] to a pooled prevalence of 60% of medical students experiencing for harassment and discrimination [59]. Among medical students, 83% of women and 41% of men experienced gender discrimination or sexual harassment [59].

In study populations of residents, 48% of residents and fellows reporting being subjected to bullying during the prior year [31] and this has been corroborated by others [74, 22, 52]. 50% of residents reported being shamed during training [54]. Some demographic groups reported an increased risk of bullying including female residents, residents aged 30 years and younger, non-white residents, and residents of a height less than 5′8″, while there was no statistical difference in rates of bullying between groups differentiated by Body Mass Index (BMI; over or under 25), Post-Graduate Year rank, international vs. U.S. medical school graduates, or U.S. citizenship status [31].

Female faculty members experienced sexual harassment at markedly higher rates than male faculty [47, 48] and perceived a gender-specific bias in the academic environment at a much higher rate than male faculty [48]. Underrepresented minority faculty and non-underrepresented minority faculty perceived and experienced racial or ethnic bias at higher rates than non-Hispanic white faculty members [60].

### Settings

Our review of the settings in which incivility occurs identified specific specialties, clinical settings, and educational settings with elevated rates of incivility. Multiple studies found higher rates of incivility in general surgery and obstetrics and gynecology [43, 59, 70]. The operating room was identified as the most common location for shame events experienced by residents, while faculty reported shaming events occurred most frequently in non-public settings [26, 33, 44, 54]. In research completed with faculty and residents, the most common location for aggressive behavior was the emergency room followed by the operating room [51]. Seventy-two percent of medical students witnessed a lapse of professionalism during clerkship versus 60% who witnessed a professionalism lapse during their pre-clerkship education; lapses of professionalism were defined in this article to include several behaviors that met the definition of incivility including arrogance, cultural or religious insensitivity, abuse of power asymmetries and bias or sexual harassment [42].

### Impact of incivility on individuals and institutional responses

Research on incivility’s impact on an individual documents effects in professional and personal domains. Among medical students, the potential professional consequences included selection of specialty [23, 70], residency rankings [70], and high burnout and depression prevalence [14, 21, 37, 41, 52, 69], while the personal effects included changes in self-image and feelings of guilt and isolation [23]. Students reported awareness of school policies on mistreatment, and they also reported low reporting rates [16]. Students’ reasons for low reporting included that the incident didn’t seem important enough to report and fear of reprisal [20, 34].

Research on the effect of incivility on residents focused on the health effects including that bullying adversely affected individuals’ health [21, 22, 31–33, 37, 71] and that disrespectful behavior toward residents was predictive of higher alcoholism screen test scores in affected residents [68]. The effect of shaming on residents included both internal reflection and self-improvement and a loss of confidence and an impact on work production [27, 54, 57, 72, 73].

For faculty, potential professional consequences of experiencing incivility included the effect on career advancement [60] and career satisfaction [29, 60] although affected faculty had similar rates of academic productivity [55, 60]. In the personal sphere, incivility resulted in diminished confidence and self-esteem for affected faculty [29, 54, 60]. Experiences of gender discrimination also increased ratings for professional isolation among faculty [29], and faculty reported internal reflection or self-improvement as the most common effect of shaming [54].

Among residents and faculty, a study revealed no significant correlation between verbal aggressiveness and the threat of legal action against physicians [51]. Residents and faculty reported increased levels of stress, frustration, reduced collaboration, reduced communication, impaired relationships, and loss of concentration as a result of disruptive behavior which was defined to include “any inappropriate behavior, confrontation, or conflict ranging from verbal abuse to physical and sexual harassment.” [65, 75]. Most importantly, disruptive behavior was also linked to impaired quality of care, medical errors, adverse events, and patient safety [22, 32, 33, 65]. Last but not least, incivility impacted the learning climate across the continuum of medical education, making learning and work difficult at all levels [27, 39, 58, 62, 67, 69, 73].

## Discussion

This study is different from previously published reports in the following aspects: 1) We present data only from North America, precluding cultural differences that likely exist in medical schools, residency programs and departments from countries outside of North America [76, 77]; 2) Although we recognize that incivility occurs across the health professions, we limited our pertinent articles to those dealing with individuals in academic health centers representing the continuum of medical education, exclusive of continuing professional education, as there was no literature addressing this aspect of education; and 3) We sought to identify specific factors that would provide more insight and detail into incivility including the perpetrators, victims, settings, types of incivility, and the impact on the individuals and department/institution.

Our study re-confirms previous studies that incivility and mistreatment across the medical education continuum is a persistent, pervasive, and often inadequately reported and addressed problem in North America. Reporting of mistreatment is mandated at the undergraduate level through the Graduation Questionnaire administered by the Association of American Medical Colleges. At the graduate level, there is no national mandate to report incivility, although there is a mechanism through an ombudsperson to report potential problems. For faculty encountering different forms of incivility, it is unclear how many medical school-affiliated departments have mandatory reporting mechanisms. Whereas the purpose of our study was to the capture detailed data on the topic published, from the articles we found, there still appears to be a great deal of underreporting, making the true prevalence of incivility difficult to determine [20, 23].

Through our review, we hoped to extract more detailed information than previous articles on the topic, in order to advance the field by identifying perpetrators, victims, and where incivility occurs. While the studies in our review did identify both victims and perpetrators in general terms, more granular descriptions of perpetrators, victims, and likely settings of incivility were not available. As an example, studies did not enumerate faculty seniority or rank, resident level, or gender information either as perpetrators or victims. The settings in which incivility occurred were not always identified, making it difficult to tailor an approach to the problem. Although our review was not able to identify detailed characteristics regarding all aspects of incivility, it may be possible to capture this data more effectively via other processes (e.g., confidential focus groups) which address specific victim groups, perpetrator groups, and/or settings.

As an overview of our study, there were determinants identified that the authors deemed significant. In terms of study design, a majority of studies used a quantitative methodology, with about 20% using qualitative methods and three studies reporting mixed methods. Based on examining study results using any of the three methodologies, none appeared advantageous regarding producing more specific data to assess the root causes of incivility. In addition, the study populations were evenly split between medical students and residents, with faculty-focused incivility representing fewer studies. Lastly, the types of incivility included vague categories such as mistreatment and more specific areas like sexual harassment, verbal and physical abuse, gender discrimination, bullying and public humiliation, among others.

The types of incivility identified in the study covered a wide spectrum amongst trainees and faculty, from verbal abuse to sexual harassment. Sexual harassment and verbal abuse occurred across the spectrum of medical students, residents and faculty and were not specific for any one group. There is another area of incivility that is less frequently identified or addressed, i.e., what the authors label as covert incivility. Covert incivility occurs when trainees are ignored, not included in team functions, and when their input is not valued as team members. This kind of incivility is more subtle and unlike verbal and physical abuse, is one of exclusion, although it can have the same effect as more overt behaviors on one’s professional development. The authors suggest that Bandura’s Social Learning theory helps to account for this perpetuation of incivility from generation to generation [13]. What is clear is that incivility is imbedded in an unsafe learning climate, resulting in a proclivity to more patient errors, thus compromising patient safety. In addition, this has led to burnout, depression, change of jobs, and to the extreme, suicidal ideation and behavior, in trainees and faculty, compromising the effective functioning of the health care team [9, 78, 79].

Because perpetrators of incivility have not been consistently identified with details regarding their gender, professional rank (e.g., intern vs junior residents; junior vs. senior faculty), age, and cultural identity, it is difficult to suggest specific prevention and remediation measures based on demographic characteristics as the target group is diverse. While perpetrators and victims of incivility were frequently linked by power differentials, three perpetrator groups were identified in the literature that may be overlooked, namely, nurses, patients, and medical school peers. While nurses have been associated with mistreatment of students, trainees, and faculty [16, 20, 31, 42, 56, 63, 40], additional data is needed to determine the kinds of incivility that have occurred and analyze the root causes of this behavior. What is clear is that the patient care model of nurses providing day-to-day patient care in both ambulatory and high acuity areas of the health system (e.g., the delivery room, the ICU, surgery and inpatient) remains reasonably stable, and trainees rotate through those areas for limited periods during their training. Anecdotally, nurses may develop a protective attitude about their ‘work turf’ through which trainees transiently pass and trainees may be inadequately prepared for work in an interdisciplinary team environment with high stress staffed by seasoned nurses.

Patients also represented an unanticipated perpetrator group, but the specific types of incivility committed by this group were not described in detail [20, 23, 31, 40]. There were notations in studies about physical and verbal abuse, sexual harassment and safety issues, but these acts were not enumerated upon. Perpetrating physician violence can certainly occur when patients are mentally unstable and under the influence of drugs. In addition, they can inappropriately and relentlessly demand tests, treatments and prescriptions from medical students, residents, and physicians, resulting in excessive counseling time, unnecessary and sometimes costly medical care, and dissatisfaction with medical care [80]. It is important to differentiate those patients that are very vocal and proactive about their care versus those who are unreasonably demanding of tests and procedures not in accordance with the standard of medical care, in addition to being inappropriate with language and physical contact.

Incivility perpetrated toward medical students by peer medical students was also reported in our search [16, 42, 51]. Incivility involving medical students as both perpetrators and victims is a disturbing finding and one that could emanate from faculty and residents modeling inappropriate behavior as well as the competitive environment of medical school; i.e., the Bandura effect. Many medical schools have adopted the AMA Code of Ethics in which chapters 9 and 10 address how physicians should interact with fellow professionals and self-regulate regarding their professional behavior [81]. Adhering to these ground rules is a reasonable expectation and deviations from the norms should be opportunities to counsel students and observe behavior change over time. There is also a study that identified resident incivility against peers [35] and the same principles for addressing this behavior applies.

Assessing the characteristics of the victims did not provide a uniform, single victim profile from our review. Whereas specific studies focused on particular groups within the continuum and suggested incivility followed the training hierarchy, most articles did not provide enough information about the victims that would be of value in the identification and approach to treatment. Additional detail regarding victims’ gender, point in training or professional rank, age, and cultural identity can help to understand the victims and determine an approach that is coordinated with the problem(s) identified. To generalize from our data, female medical students were more likely to be victims of incivility than males, fellows and residents were likely to be bullied, under-represented minorities were more likely to be on the receiving end of racial or ethnic bias versus non-Hispanic whites, and females were more likely to be sexually harassed than males.

The reported settings of incivility were diverse enough that it is not possible to pinpoint specific settings in which incivility occurs. Some departments and clinical settings (e.g. obstetrics and gynecology, surgery, operating room, emergency room) seem to report higher rates of incivility but the underlying reasons, i.e., root causes, for this remain unclear. Further research might help identify factors that cause higher rates of incivility in these areas and determine if they are associated with the culture, fast-paced environment, and high-risk decision-making. If indeed incivility behaviors occur in specific areas, these could then be the focus for further data collection in terms of if there are embedded cultural issues, personnel, or structural issues that might be contributing to this problem.

It is evident that incivility has been reported to have had a significant effect on individuals, including health effects [31, 68], diminished self-confidence and self-esteem [23, 29, 54, 60], and burnout and diminished career satisfaction [14, 29, 60, 82]. Medical students report a high burnout rate, isolation, alcohol abuse and think differently about their career choice when they are the subjects of incivility. The burnout phenomenon is not new to medicine as one traverses the arduous task of formal education and clinical training over years. However, there is recent evidence that burnout can result from a learning climate that is not safe, especially when there is incivility inherent in the culture. This also has been shown to pose a risk for patient safety and increased medical errors. Not tolerating incivility and teaching trainees and faculty what the characteristics are of a safe learning climate will be helpful in changing this culture. Lastly, and not surprisingly, faculty report leaving their academic jobs or in fewer instances, clinical medicine altogether, because of adverse work climate issues that impact their satisfaction and performance [83, 84].

The institutional impact has been reported infrequently but we noted institution-wide changes in how incivility is reported and the creation of guidelines that address how the organization seeks to identify this behavior and then how to deal with it [16, 20, 65, 70, 40]. The LCME guidelines specifically address medical student mistreatment and challenge medical schools to set up a reporting and remediation process to confront incivility as residents and faculty interact with students [85]. It is too early to determine the effects of these programs as students may still not feel empowered to confront the hidden curriculum that has permeated the medical culture for so long [12]. The Accreditation Council for Graduate Medical Education (ACGME) also requires programs to ‘identify resident mistreatment’ but there does not appear to be any national repository to collect data to assess the prevalence of this problem [86]. The Joint Commission has also mandated that academic health centers have processes in place to manage disruptive behavior [10]. The possible compromise of patient care, decreased work productivity, discordance of team functioning and faculty (and trainees less so) leaving their jobs all have a clear-cut impact on the institution.

Whereas bullying or incivility in school-aged children, politics, and in the workplace makes headlines, the incidence and ultimate impact appear to be underreported in medical education [16]. Underreporting may be due to fear of consequences on the part of the victim, an ingrained culture of negativity towards those lower in the medical education hierarchy, and perhaps because this behavior has been tolerated and accepted due to power differentials between perpetrators and victims. Another possible cause for underreporting is the subjectivity of the matter. Incivility investigations often result in a difference of opinions, unless another outside witness can verify events, which changes the anonymity of the process. In previous years, intervention programs based on education and anonymous reporting systems were established at institutions in the United States, with little to no decrease in mistreatment [40]. Anonymous reporting makes verification of events nearly impossible, but identified reporting perpetuates the fear of retaliation. This makes interventions that much more of an obstacle. It also makes generalization and application of systems for change rather difficult as well.

One of the barriers and limitation of the study was that the authors encountered numerous and varied terms; i.e., keywords, used in the literature to identify studies on incivility. Consequently, searching the literature for evidence and pervasiveness of incivilities was a challenge. Based on our experience reviewing the literature, we recommend using ‘incivility’ to describe all forms of mistreatment across the continuum of medical education for more clarity in further research. Incivility is a broader term that includes any behavior within the field that negatively affects the individual, team and/or institution. Based on this scoping review, the authors suggest that the topic of incivility has been investigated and addressed enough, with newer publications not adding additional information to the existing literature in terms of addressing the problem. It is disheartening to see the same incivilities reported over and over again, with too-often recommendations made that further studies need to be done to define the problem. Intervention systems and models will only be pursued when incivility in medical education is seen as a priority issue to be addressed.

The reporting of incidents should not imply there will be punitive action against individuals; instead, we should seek educational interventions to make the learning environment safer, e.g., mandating workshops for faculty, staff and trainees on the characteristics of a safe learning climate, with a special focus on the teacher-learner, provider-patient, and health care professional to health care professional relationships. Content should include burnout, patient safety, hierarchical relationships, and institutional and individual impact.

Incorporating communication skills training into the curriculum at the undergraduate and graduate levels that addresses inappropriate and aggressive patient behavior towards trainees should be considered for teaching trainees how to recognize the problem and negotiate with these patients. Patient abuse of trainees is not acceptable, and educators should incorporate techniques into the curriculum that make boundaries clear and provide trainees with response strategies.

With peer-on-peer incivility not acceptable, educational interventions also need to be directed towards that problem. Students have been the products of a competitive environment from the beginning of their education in which attaining the highest levels has been the benchmark for realizing medical school acceptance and residency selection. Course directors, residency training program and clerkship directors, in addition to educational deans should proactively address unacceptable behavior that has been noted in peer-on-peer incivility in the literature. Orientation of trainees at all levels that specifically address peer-on-peer incivility and state a zero-tolerance policy with significant consequences should be an integral part of our educational culture.

The authors suggest that educators consider creating interactive workshops incorporating role-play or simulated scenarios that address the workings of interprofessional/interdisciplinary teams (nurses, allied health professionals, and the hierarchy inherent in teams) to decrease incivility in the clinical setting. Our data was specific to nurse abuse of trainees but expanding education to the training involving other health professionals would also be proactive in identifying and dealing with these incidents [87]. These are teachable behaviors and based on our findings, should be part of the training across the continuum of education.

Finally, incivility is not tolerated in the workplace in some countries, the U.S. being an exception [88]. The authors suggest that this approach be considered for the continuum of medical education, reducing the incidence of what all would agree is unacceptable behavior in a learning environment that needs support for learners at all levels. Emphasizing what should not be tolerated in the workplace as opposed to making exceptions for physicians and other healthcare providers because they create revenue or are looked upon as favored employees is critical, i.e., zero-tolerance regulations. Whereas further studies need to be completed to establish details that our study was not able to extract, academic centers and national accrediting organizations should be looking to change the climate by establishing national standards in the workplace. Starting with establishing transparency regarding acceptable and unacceptable behaviors and making it clear that there is a zero-tolerance policy on incivility should be uniform policy across academic health centers. Medicine has tolerated this unacceptable behavior long enough and responsible leadership need not wait longer to improve the health care environment for our trainees, faculty, and patients.

## Conclusion

This study contributes a number of innovations to the literature: 1) it is a scoping review across the continuum of medical education in North America; 2) it has suggested that the term 'incivility' should encompass all forms of mistreatment for future studies 3) it has sought root causes of incivility in the stakeholders; and 4) it has identified perpetrators that have not always been on the forefront of the topic, e.g., one-on-one student, patient and nurse incivility.

Incivility across the continuum of medical education is pervasive and further studies will not change this culture. This study was unable to discern details on the characteristics of learners and teachers regarding incivility. The authors suggest that establishing a safe learning climate through transparent faculty, resident, and student orientations, clear curricular goals and objectives, and development of educational interventions that promote safe learning climates are sound and proven methods to address this issue [89]. Finally, because addressing cultural change in academic medical centers has been challenging, implementing zero-tolerance standards and policies at national and local levels should be a priority.

## Data Availability

PubMed search strategy and articles that met inclusion criteria are included in this manuscript.

## Appendix

### Example search strategy used to retrieve relevant journal articles

Database: PubMed

("Bullying"[Mesh] OR "Aggression"[Mesh] OR "Sexual Harassment" OR mistreat* OR bully* OR harassment[title] OR belittl*[title] OR civility[title] OR incivility[title] OR "medical student abuse" OR intimidation[title] OR “disruptive behavior*”[title] OR “Interpersonal conflicts”[title] OR “adverse experiences”[title] OR “psychological abuse” OR (discriminat*[title] AND (gender[title] OR race[title] OR ethnic*[title] OR gay[title] OR lesbian[title] OR GLBT[title] OR bisexual*[title] OR sex[title])))

AND

(("Students, Medical"[Mesh] OR "Education, Medical"[Mesh] OR Internship and Residency[MeSH] OR "Schools, Medical"[Mesh]) OR "medical student*" OR (medical[title] AND student*[title]) OR (medical[title] AND education[title]) OR ((resident[Title] OR residents[Title] OR residency[Title]) AND (education or training)) NOT (“nursing home” OR “nursing homes” OR “assisted living”))

Filters: English

Filters: 2000-Present

## Abbreviations

ACGME: Accreditation Council for Graduate Medical Education
BMI: Body Mass Index
MeSH: Medical Subject Headings
